# Demonstrating the Viability of Spiritual Care Education: A Pilot Study on Integrating Spirituality and Health into Medical Education

**DOI:** 10.1177/23821205251336846

**Published:** 2025-05-15

**Authors:** Haafiz Hashim, Zuhair Zaidi, Ahmed Alshaikhsalama, Ammaar Kazi, Zaiba Jetpuri

**Affiliations:** 1 12334 University of Texas Southwestern Medical School, Dallas, TX, USA; 2 Department of Family and Community Medicine, 12334 University of Texas Southwestern Medical Center, Dallas, TX, USA

**Keywords:** holistic care, medical education, spiritual care, religion, spirituality

## Abstract

**Background:**

Despite the recognized significance of integrating spiritual care into healthcare, training in spiritual care is often an elective rather than a core component of medical education in the United States, suggesting a gap in the comprehensive training of future healthcare professionals.

**Methods:**

An elective course was developed to explore the interplay between religion, spirituality, and medicine and was administered over 2 academic semesters. The curriculum included lectures, interactive sessions with religious leaders, and class discussions aimed at enhancing understanding and implementation of spiritual care. Course efficacy was evaluated using pre- and postcourse assessments quantifying student aptitudes and attitudes toward spiritual care. The second semester also included a comparison group that was not enrolled in the course, matched to the enrolled students on the basis of age, gender, and religiosity.

**Results:**

A total of 19 medical students voluntarily participated over 2 semesters. Semester 1 students demonstrated modest nonsignificant increases in attitudes toward spiritual care. Semester 2 students exhibited increased interest, understanding, and perceived ability to provide spiritual care. Furthermore, semester 2 aptitude scores increased from 51% to 78%, demonstrating significantly improved ability to navigate spiritual care case scenarios. While students enrolled in the course had improved postcourse survey scores, the comparison group of students that did not enrol in the course had no change in their pre and postcourse surveys.

**Conclusions:**

This elective course successfully addressed a gap in medical education by improving student aptitudes and attitudes toward spiritual care. The course model offers a framework for other medical schools aiming to enhance spiritual care training, underscoring the need for medical curricula to prepare well-rounded healthcare professionals capable of providing holistic patient care.

## Background

In recent years, the intersection of religion & medicine has garnered significant attention, highlighting the need for a holistic approach to patient care that encompasses not only physical but also spiritual well-being.

Understanding the distinction between religiosity and spirituality is essential in this context. Religiosity refers to an individual's adherence to the beliefs, practices, and rituals associated with an organized religion. It encompasses behaviors such as attending religious services, participating in communal worship, and following specific doctrinal teachings.^1^ In contrast, spirituality is a broader, more personal pursuit of meaning, purpose, and connection, which may or may not be linked to a particular religious tradition. It reflects an individual's intrinsic beliefs and experiences concerning the transcendent or sacred. Recognizing this distinction is crucial for healthcare providers. By appreciating the unique spiritual and religious perspectives of each patient, practitioners can offer more personalized and respectful care, thereby enhancing the overall well-being of those they serve.

Numerous studies have demonstrated that addressing religious and spiritual beliefs significantly impacts health outcomes. For instance, a systematic review of 43 studies found that patients who received spiritual care reported better quality of life, improved emotional well-being, and reduced levels of anxiety and depression. Additionally, incorporating patient spirituality into medical care has been linked to better adherence to treatment plans and enhanced coping mechanisms during illness.^2^ A 2018 study revealed that 70% of patients desired their physicians to consider their spiritual needs, emphasizing the importance of religious understanding in healthcare practices.^3^ Hospitals that integrate chaplaincy services and spiritual support show lower rates of hospital readmission and shorter lengths of stay, ultimately contributing to cost savings.^4^ Furthermore, religious and spiritual support have been shown to improve end-of-life care, with patients experiencing less aggressive medical interventions and more meaningful and peaceful final moments.^5^ Thus, it is apparent that training physicians in spiritual care principles is vital to improving the patient experience and improving health outcomes.

However, despite extensive evidence of the effectiveness of spiritually informed care, proper training in addressing patient religion and spirituality (R/S) needs remains lacking. While over 90% of US medical schools address spirituality and health (S&H) within their curriculum, the subject remains predominantly elective or addressed only briefly in a piecemeal fashion among other topics. This is despite the fact that discussions of spirituality are perceived by trainees to be an advanced communication skill, requiring greater expertise compared to other tasks such as discussing “do not resuscitate” orders with patients.^6^ This lack of formal spiritual care training has a direct effect on implementation in medical practice; one study examining an S&H curriculum among internal medicine residents demonstrated that the primary obstacle to effective spiritual care in residents is a lack of knowledge regarding the fundamentals of addressing patient spirituality.^7^ This lack of spiritual care competence persists after residency; a nationwide survey of practicing attending physicians revealed that half of all physicians never talk to their patients about spirituality.^8^ Analysis of associated factors demonstrated that exposure to S&H training in medical school was uncorrelated, indicating that current training methods are inadequate for training physicians to addressing patients’ spiritual needs.^8^

One major cause for the paucity of spiritual care education in medical pedagogy is a lack of evidence-based training methodologies. Promising models have been described in studies such as that by Vitorino et al but are limited by a lack of quantitative learning assessments demonstrating effectiveness.^9,10^ This dearth of proven S&H educational curricula has compounded low interest among medical schools in expanding existing training; while the majority of medical school deans acknowledge the necessity of effective spiritual discussion in patient care, only 25% are prepared to expand training on addressing patient spirituality.^10^

Thus, this study has 2 main objectives. The first is to investigate training approaches that are effective in training S&H to medical students. The second is to encourage further investigation into S&H training by proving the viability of developing a quantifiably effective spiritual care curriculum.

## Methods

This was a prospective cohort study performed at the University of Texas Southwestern (UTSW) Medical School aimed at examining the efficacy of our proposed S&H curriculum. The first semester was purely observational, while the second semester also included a comparison group. Our investigation abides with the DocTRINE guidelines for reporting innovation in medical education (see Appendix E).^11^

### Course Objectives

Our course was designed to educate students on the interplay between religion, spirituality, and medicine, with the goal of equipping medical students with the skills to provide spiritually informed holistic patient care.

Our course design was guided by 2 foundational objectives:

#### Nurturing Student Attitudes Toward Spiritual Care

The first goal of our curriculum was to improve student attitudes toward spiritual care. Attitudes, or internal states of a person toward anything that a person can evaluate, have been demonstrated to be closely associated with overall achievement and motivation.^12^ In our case, we hoped to improve student attitudes toward spiritually informed care in order to motivate them to further refine and practice their skills in future patient care. Our primary approach was to expose students to individuals from a variety of roles in the healthcare system, including patients, family members, chaplains, nurses, physicians, and faith leaders to help them understand the multifaceted role that R/S play in health and wellbeing. We also hoped to instill in students an appreciation for the diversity of human spirituality and religious thought by including instructors from the entire spectrum of religious and spiritual (or nonspiritual) backgrounds.

#### Enhancing Student Aptitude in Spiritual Care

The second major focus of our curriculum was to improve students’ aptitude for spiritually informed care. Aptitude encompasses all cognitive, conative, and affective characteristics needed for success in learning or performance and is generally used to signify student preparedness.^13^ Thus, our course aimed to help students expand their ability to grasp, analyze, and apply the principles needed to address the nuanced aspects of S&H. To further this goal, we identified core competencies that students should master, including knowledge of major belief systems, patient communication skills, medical ethics, and spiritual care resources. Through case studies and practice sessions with real patients and family members, students were challenged to apply these competencies in an empowering and nonjudgmental environment, developing their proficiency and readiness to utilize their skills.

### Course Implementation

This course was implemented at the UTSW Medical School over 2 semesters, from January to December 2023. The course was offered on a purely elective basis, with no impact on students’ grades; thus, no exclusion criteria were applied. The course was offered in a hybrid educational format, with both online and in-person sessions. Outside of recruiting a varied team of educators, the resources required for implementing the course were minimal.

The semester 1 cohort consisted of 8 students and was led by 2 upperclassman medical students with faculty supervision. The course focused on the 5 major world religions and nontraditional spiritual perspectives, utilizing a didactic lecture format followed by case-based discussions and application questions. While efforts were made to diversify the instructors as much as possible, logistical limitations meant that most instructors represented the Abrahamic faiths (Christianity, Judaism, and Islam), although some Hindu, Buddhist, and nontraditional spiritual perspectives were explored in practice sessions.

The semester 2 cohort consisted of 11 students and was led primarily by 2 students who had taken the course in the first semester. Based on verbal feedback provided by semester 1 students, this version of the course aimed to reduce the role of didactic lectures and place a greater emphasis on discussion-based learning led by experienced professionals from the healthcare system and the broader community. Expanding on the efforts taken in semester 1, instructors for semester 2 were drawn from a diverse variety of spiritual backgrounds, including all 5 major world religions as well as practitioners of nontraditional spirituality and less common religious/spiritual backgrounds. A variety of roles within the healthcare system were also explored, with patients, family members, physicians, chaplains, and community leaders all represented among the educational team. Discussions were followed by application-based sessions featuring ethical scenarios as well as weekly assessments to encourage further reflection and mastery of core competencies. Due to the elective nature of the course and low overall student numbers, randomization of the study participants was not possible.

### Study Design

To determine the overall efficacy of the course, data were collected from students in the form of pre- and postcourse surveys. Because no validated surveys exist for spiritual care education, we developed our own assessments for both spiritual care attitudes and aptitudes. For the first semester, the surveys consisted of 6 items: 2 items assessing student religiosity and spirituality, and 4 items meant to gauge attitudes toward core components of spiritually informed care. These attitude assessment items were formatted in the form of statements encompassing different components of spiritually informed care, with students asked about their attitude toward the statement via a 5-point Likert scale (rating their agreement from “strongly disagree” to “strongly agree”). This type of scale is commonly employed in medical education for purposes such as end-of-rotation trainee feedback, faculty evaluations of trainees, and assessing performance after educational interventions.^14^ Attitude survey items are outlined in Table 1.

**Table 1.** Semester 1 Attitude Assessment.

How would you rate your level of religiosity? (1 least religious—10 most religious)
How would you rate your level of spirituality? (1 least spiritual—10 most spiritual)
How comfortable do you feel treating a patient from your own religion? (1 least comfortable—5 most comfortable)
How comfortable do you feel treating a patient from a different religion? (1 least comfortable—5 most comfortable)
Rate this statement (1 strongly disagree—5 strongly agree): Considering religious beliefs is a necessary component of patient care.
Rate this statement (1 strongly agree—5 strongly disagree): it is important to have spiritual leaders (eg, priest, imam, chaplain, rabbi) on staff.

For the semester 2 attitude assessment, statements were graded on a 10-point scale to allow for finer differentiation of attitudes. Furthermore, the number of items was reduced to encourage reliable survey completion. Questions relating to religiosity and spirituality were combined and the remaining items were changed to focus on the first 3 levels of the Kirkpatrick model of effective training: reactions, learning, and behavior. The semester 2 attitude assessment can be seen in Table 2.

**Table 2.** Semester 2 Attitude Assessment.

On a scale of 1-10, with 10 being the best, how interested are you in exploring scenarios where medical decisions intersect with spirituality and religion?
On a scale of 1-10, with 10 being the best, how would you rate your overall understanding of religion, spirituality, and medicine?
On a scale of 1-10, with 10 being the best, rate your ability to engage in discussions with patients about spirituality and religion in the context of medical care.
On a scale of 1-10, with 10 being most religious, how would you rate your level of spirituality/religiosity?

In addition to attitude testing, we implemented a 10-item aptitude assessment in semester 2, designed to test students’ knowledge, analysis, and application of core competencies addressed in class. Because no validated assessments exist for spiritually informed care education, our course instructors designed a multiple-choice assessment, including scenarios incorporating a broad range of religious and spiritual traditions. Most questions focused on medico-ethical reasoning in the context of the religious/spiritual patient, with an emphasis on knowing when to access spiritual care resources. Notably, core skills such as counseling patients and taking a spiritual history were not included in the survey despite being a major part of the course due to the patient-specific nature of such discussions and the difficulty of designing effective multiple-choice questions testing such concepts in a standardized way. Assessment of such skills was captured in our course through in-person feedback (not included in this study) and attitude survey items gauging student confidence. Aptitude survey items are outlined in Table 3.

**Table 3.** Semester 2 Aptitude Assessment.

Sarah, a 28-year-old woman, is admitted to the hospital due to severe hemorrhage following a car accident. The medical team determines that a blood transfusion is necessary to save her life. However, Sarah is a Jehovah's Witness, and declines the transfusion. What should the medical team do in this situation? **a) Respect Sarah's religious beliefs and withhold the blood transfusion.** b) Administer the blood transfusion regardless of her beliefs to save her life. c) Consult with the hospital's legal team to force the blood transfusion. d) Persuade Sarah to change her religious beliefs to accept the transfusion.
Ahmed, a 45-year-old Muslim man, has type 2 diabetes and needs to take his medication regularly and eat a balanced diet. However, Ahmed is observing fasting during Ramadan, refraining from eating or drinking from sunrise to sunset. What should Ahmed's physician consider when managing his diabetes during Ramadan? a) Advise Ahmed to skip fasting for medical reasons. b) Suggest Ahmed takes his medication during the fasting period. **c) Schedule appointments during the evening to accommodate fasting.** d) Ignore his religious beliefs and insist on a regular eating schedule.
David, a 10-year-old boy, is diagnosed with a treatable condition, but his parents are devout Christian Scientists. They refuse medical care and insist on using prayer as the sole form of treatment. How should the healthcare provider navigate this situation? a) Respect the parents’ wishes and rely on prayer as treatment. b) Administer medical treatment against the parents’ wishes for the child's benefit. c) Report the parents to child protective services for neglect. **d) Consult with a religious leader to mediate the disagreement.**
Maria, an elderly woman, is in the final stages of cancer. She is a devout Buddhist and is adamant about not using pain medication, as it is believed to cloud the mind and disrupt the transition to the afterlife. On physical exam, Maria is in excruciating pain. How should the medical team handle Maria's pain management in consideration of her religious beliefs? a) Administer pain medication against her wishes to alleviate suffering. b) Respect Maria's religious beliefs and withhold pain medication. **c) Consult with a religious leader to help make the final decision.** d) Transfer Maria to a different facility that aligns with her beliefs.
John is admitted to the hospital with a severe, life-threatening health condition. The primary treatment for John's condition is derived from a certain food item; however, John's personal spiritual beliefs forbid him from consuming it under any circumstances. How should the healthcare team navigate this challenging situation, given John's life-threatening condition and the conflict with his spiritual beliefs? a) Respect John's beliefs and refrain from administering any treatment involving the food item. **b) Attempt to educate John about the necessity of medical treatment while respecting his beliefs.** c) Consult with a medical ethicist to mediate the discussion between the healthcare team and John. d) Seek a court order to authorize medical intervention, even if it involves the prohibited food item.
John is urgently in need of a heart transplant. However, he refuses to receive a heart from a donor of a different religion. How should the medical team address John's concern? a) Assure John that the donor's religion doesn't affect the success of the transplant. **b) Respect John's request for a donor with a similar religious background.** c) Administer the transplant without disclosing the donor's background. d) Persuade John to accept the transplant, regardless of the donor's religion.
Leila, a Muslim girl, requires surgery on her leg. She wishes to hold a Quran during the procedure to maintain her religious connection. What should the medical team do to accommodate Leila's religious beliefs during the surgical procedure? a) Refuse to allow Leila to hold the Quran as it compromises the sterile field. **b) Respect Leila's request and perform the surgery with the Quran nearby.** c) Have a different provider perform the surgery due to your dislike of religion. d) Agree to respect Leila's beliefs but remove the Quran before surgery to maintain sterility
A group of parents in a predominantly Orthodox Jewish community refuse to vaccinate their children due to religious beliefs, contributing to an outbreak of a vaccine-preventable disease at a local elementary school. How should healthcare providers address this issue? a) Respect the parents’ religious beliefs and allow unvaccinated children to attend school. **b) Work with community leaders to educate and encourage vaccination.** c) Deny medical care to unvaccinated children as a public health measure. d) Report the parents to child protective services for endangering their children.
Michael is terminally ill with no chance of recovery. He believes deeply in the sanctity of life and would make every effort to extend his life, even if it means prolonging his suffering. He requests an unproven, aggressive procedure with a very low chance of success. How should the healthcare team approach Michael's request? a) Administer aggressive treatment as requested, regardless of the prognosis. b) Provide palliative care to alleviate suffering. **c) Consult with the hospital's spiritual care team to discuss Michael's wishes.** d) Persuade Michael to abandon his religious beliefs and accept the prognosis.
Liam, a 5-year-old boy, is admitted to the hospital with a life-threatening illness. His parents are Hindus who believe that rituals involving chanting and incense must be performed at the bedside daily to ensure he recovers. How should the medical staff handle the parents’ request? **a) Allow the parents to conduct the rituals with supervision at Liam's bedside.** b) Forbid any religious rituals in the hospital, as it may disrupt care. c) Consult with a religious leader to mediate the decision. d) Transfer Liam to a different facility that accommodates such requests.

For semester 2, we included a comparison group to ensure that changes in attitude and aptitude could be attributed to the course. Students who had been on the course waitlist were asked to join the comparison group, completing course surveys at the same time as the enrolled students. To minimize the risk of selection bias, both groups were matched based on religiosity, spirituality, age, graduating class, and gender. Analysis revealed no significant differences between groups, confirming the representativeness and diversity of the sample across both cohorts.

### Statistical Analysis

Statistical analysis was conducted to evaluate changes in student aptitude and attitude scores, with comparisons made both within and between groups. Paired Student *t* tests were utilized to compare pre- and postcourse assessments within each cohort, while unpaired Student *t* tests were employed for comparisons between the experimental (Fall 2023 cohort) and comparison groups.

Due to the elective nature of the course and limited available enrollment, no preliminary power analysis was carried out. As this is an exploratory study meant to establish the feasibility of implementing spiritual care education within a medical curriculum, no applicable estimates of expected effect size were readily available. As such, the study was not designed to achieve specific statistical power but rather to provide foundational insights and inform future research in this area.

### Ethical Considerations

The research adhered to ethical standards for human subjects, with voluntary participation and measures to ensure anonymity and confidentiality of responses. Survey results and participants had no impact on the medical students’ passing or grade in the class. The study protocol received approval from the UT Southwestern Medical Center Human Research Protection Program, ensuring ethical integrity and participant privacy protection.

## Results

A total of 19 students participated over 2 semesters: 8 students (4 male, 4 female) enrolled in semester 1, including 6 first-year, 1 second-year, and 1 third-year medical student; 11 students (5 male, 6 female) enrolled in semester 2, including 10 first-year medical students and 1 second-year medical student. For semester 1, 2 students did not complete the course, resulting in 6 total students (3 male, 3 female) submitting both pre- and postcourse surveys. For semester 2, all 11 students completed the course and submitted both surveys. Our comparison group consisted of 11 (6 male, 5 female) first-year medical students who took both pre- and postcourse surveys without enrolling in the course. All surveys were completed on a voluntary basis and extrapolated to a 10-point scale.

### Attitude Testing

Attitude data showed improvements in both semesters compared to precourse responses. The results for attitude assessments of semester 1 students are summarized in Table 4. Increases were noted in all 4 items assessing student attitude, especially comfort in treating patients from a different religion (5.8 vs 6.8; *P* = .52) and considering religious/spiritual beliefs as a necessary component of spiritual care (6.1 vs 7.4; *P* = .44). While we were able to appreciate modest improvements in student attitudes during semester 1 of the course, no statistically significant changes were detected.

**Table 4.** Semester 1 Student Attitude Assessment Results (n = 6).

Parameter	Precourse average rating	Postcourse average rating	*P* value
Level of religiosity	6.4	6.3	.99
Level of spirituality	7	7.4	.86
Comfort in treating a patient from the same religion	7.3	7.9	.83
Comfort in treating a patient from a different religion	5.8	6.8	.52
Considering religious beliefs as a necessary component of care	6.1	7.4	.44
Perceived importance of having spiritual leaders on staff	6.4	7.3	.61

Results for attitude assessments of semester 2 students are summarized in Table 5. For this iteration of the course, we were able to detect statistically significant improvements in all 3 attitude assessment items. The magnitude of change was also increased compared to semester 1, with an average increase of 2.5 points per survey question (not counting the item testing religiosity) compared to a change of 0.95 points per item in semester 1. Interestingly, we also detected a significantly increased score for the religiosity survey item despite this not being an aim of the course.

**Table 5.** Semester 2 Student Attitude Survey Results (n = 11).

Parameter	Precourse average rating	Postcourse average rating	*P* value
Interest in spirituality, religion, and medicine	6.8	9.4	**<.01**
Understanding of religion, spirituality, and medicine	6.5	9.2	**<.01**
Perceived ability to have discussions with patients about spirituality/religion	7.3	9.5	**<.01**
Self-perceived religiosity	6.8	8.9	.**02**

Emphasizes significant *P* values in bold.

Data from our semester 2 comparison group are summarized in Table 6. No significant differences were noted between the pre- and postcourse surveys. Interestingly, there were nonsignificant reductions seen in all 4 survey items.

**Table 6.** Comparison Group Attitude Survey Results (n = 11).

Parameter	Precourse average rating	Postcourse average rating	*P* value
Interest in spirituality, religion, and medicine	6.3	5.7	.34
Understanding of religion, spirituality, and medicine	6.8	5.8	.07
Perceived ability to have discussions with patients about spirituality/religion	5.6	5.5	.75
Self-perceived religiosity	6.3	5.4	.16

Data comparing the precourse surveys of our semester 2 students and the comparison group are summarized in Table 7. The enrolled students did have a significantly higher perception of their ability to have discussions with patients about spirituality/religion (7.3 vs 5.6, *P* = .04). No significant differences were noted between the students and the comparison group for the other survey items.

**Table 7.** Precourse Attitude Survey Results for Semester 2 Students (n = 11) and Comparison Group (n = 11).

Parameter	Enrolled students average rating	Comparison group average rating	*P* value
Interest in spirituality, religion, and medicine	6.8	6.3	.46
Understanding of religion, spirituality, and medicine	6.5	6.8	.63
Perceived ability to have discussions with patients about spirituality/religion	7.3	5.6	.**04**
Self-perceived religiosity	6.8	6.3	.53

Emphasizes significant *P* values in bold.

Data comparing postcourse survey results between our semester 2 students and the comparison group are summarized in Table 8. Enrolled students showed significantly increased attitudes versus the comparison group in all 3 attitude items (interest, perceived understanding, and perceived ability to discuss spirituality/religion) on the survey. Differences between enrolled students and the comparison group were pronounced, with *P* values <.00001 for all attitude items. In addition, enrolled students demonstrated significantly increased self-perceived religiosity compared to the comparison group (9.4 vs 5.7, *P* < .001). Overall, our results demonstrate strong changes in attitude among enrolled students that can likely be attributed to the course.

**Table 8.** Postcourse Attitude Survey Results for Semester 2 Students (n = 11) and Comparison Group (n = 11).

Parameter	Enrolled students average rating	Comparison group average rating	*P* value
Interest in spirituality, religion, and medicine	9.4	5.7	**<.001**
Understanding of religion, spirituality, and medicine	9.2	5.8	**<.001**
Perceived ability to have discussions with patients about spirituality/religion	9.5	5.5	**<.001**
Self-perceived religiosity	8.9	5.4	**<.001**

Emphasizes significant *P* values in bold.

### Aptitude Testing

**Figure 1.**
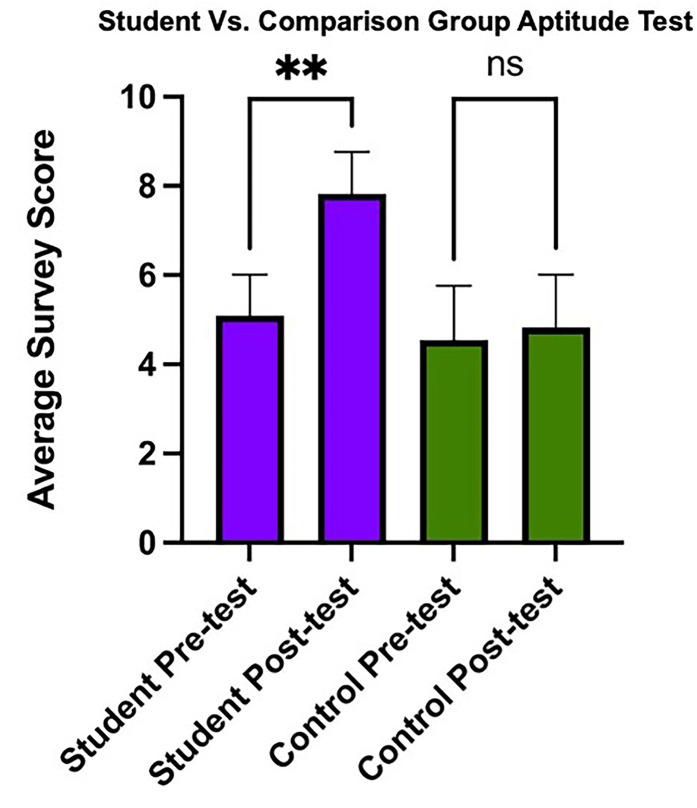
Pre- and Posttest Aptitude test Scores for 22 Participants (11 Comparison, 11 Student Group) Show a Significant Increase for Students Following Course Completion (in Purple), Compared to Nonsignificant Improvement (ns) in the Comparison Group (in Green). The *y*-Axis Represents Mean Scores with Error Bars Indicating the 95% Confidence Interval.

Aptitude assessment results for semester 2 are summarized in Figure 1. No significant differences were noted at the start of the course between enrolled students and the comparison group (48% vs 51%; *P* = .44). Following completion of the course, the student group had significantly improved scores compared to the precourse evaluation (51% vs 78%; *P* < .001). The enrolled students also significantly outperformed the comparison group on the postcourse aptitude assessment (78% vs 48%, *P* < .001). No appreciable improvement was noted in the comparison group during the same period (45% vs 48%; *P* = .91).

## Discussion

Over the 2023 to 2024 academic year, our “Religion and Medicine” course was able to demonstrate quantifiable improvements in student attitudes and aptitudes toward S&H. While semester 1 showed modest attitude improvements, our methodology hindered our ability to draw conclusions. This led us to implement major changes in semester 2, allowing us to appreciate significant improvements in both aptitudes and attitudes toward S&H. Furthermore, by comparing the curricula and course delivery between semesters, we were able to identify effective pedagogical techniques for teaching effective spiritual care to medical students. Overall, we believe that our course provides an effective template for further investigation of S&H training curricula.

### Course Efficacy

#### Course 1

While course 1 showed promising increases in student attitude toward spiritual care topics, the changes were not statistically significant, likely due to several methodological and logistical issues. Due to the elective nature of the course, the class size was limited to 8 students. Combining this with the attrition rate of 25% meant that conclusions were based on the survey results of only 6 students. This low sample size meant that our study was relatively underpowered, making it difficult to draw any statistically meaningful conclusions from the data. Furthermore, our 6-item survey only assessed student attitudes and impressions toward spiritual care, thus only capturing the first level (reaction) of Kirkpatrick's model for assessing effective training curricula. Because of these shortcomings, we decided to implement significant changes to the study methodology in order to better capture changes in student understanding in course 2.

#### Semester 2

Several important changes in our methodology meant that we were able to draw much more robust conclusions from semester 2. Most important was the addition of a 10-item aptitude assessment which tested students on concepts addressed throughout the course, including communication skills, spiritual care resources, knowledge of world religions, and medical ethics. This allowed us to identify increases in student aptitudes in addition to attitudes, making it possible to capture the second level (learning) of Kirkpatrick's model. Another major difference was the implementation of a comparison group in order to mitigate selection bias. This group of students was selected from the same academic year as most of the students in the course and items assessing student self-perception of religiosity and spirituality were used to make sure that religious and spiritual commitment was similar between groups. Prior to course administration, no significant differences in religiosity or spirituality were noted; however, after the course, we noted slight increases in religiosity among enrolled students and slight decreases among the comparison group. While this was not an aim of the course, these differences may be a consequence of improved attitudes toward R&S noted among enrolled students. Finally, our course size nearly doubled from 6 to 11, improving the statistical power of our survey. These changes allowed us to identify marked improvement in student understanding of S&H, with statistically significant changes in both aptitudes and attitudes toward spiritual care.

### Teaching Methodology

Our training approach also changed significantly between courses 1 and 2. During the first semester of the course, our primary emphasis was on improving students’ knowledge of spiritual care principles through didactic lectures. However, this led to poor engagement among students and a high attrition rate, with student feedback asserting that the information was difficult to retain. To mitigate this issue, we pursued a primarily discussion-based approach in the second course, aiming to teach concepts to students through guided questioning and case examples. This was much better received by students, who noted that the case examples in particular were especially helpful in practicing the application of spiritual care competencies. Case-based learning used in such a supplementary role has been demonstrated to improve aptitude and readiness for applying concepts to real patient cases.^15^ Other benefits of this methodology include better attendance and increased favorability among students compared to traditional didactic instruction, both of which were vital due to the elective nature of our course.^16,17^

Another approach we utilized was guided practice through a session focused on spiritual counseling. This session saw students involve themselves in discussions with real patients and family members who had undergone a variety of difficult clinical situations such as losing a loved one, end-of-life decision-making, pregnancy termination, and hospice care. Verbal feedback provided immediately after the session indicated that students felt that this opportunity to practice in a judgment-free environment made them feel better able to implement these skills in future patient encounters. These findings are supported by literature, which shows that talking to real or standardized patients allows for better learning of communication skills. This effect is especially notable for patient-centered skills such as counseling and asking questions, both of which are vital in effective spiritual care.^18^ While we did not grade student performance in these practice sessions, future iterations of the course could assess these interactions and provide feedback to students, thereby capturing level 3 (behavior) of Kirkpatrick's model. By including different evidence-based educational techniques and providing opportunities to practice S&H competencies, we were able to create an effective yet flexible framework for teaching spiritual care.

### Strengths and Limitations

Given the lack of established evidence-based spiritual care curricula, our study was primarily exploratory, aiming to prove the viability of such an undertaking. In that respect, it has several strengths which should be acknowledged. First, the inclusion of questions assessing both attitude and aptitude allowed us to capture level 2 of Kirkpatrick's model of effective training regimens, a marked improvement over similar projects which focus primarily on student attitudes and perceptions (level 1). Second, the inclusion of a comparison group validated our findings and increased the generalizability of our conclusions. By matching the comparison group and enrolled students on the basis of religiosity, spirituality, age, and gender, we further ensured that our results were due to our curriculum and not selection bias. Third, our administration of 2 separate courses allowed us to learn from the shortcomings of semester 1 and make improvements to both the curriculum and methodology of semester 2. Fourth, our course curriculum was a major strength, involving varied educational approaches and educators from numerous roles within the fields of healthcare and spirituality, including physicians, nurses, patients, family members, hospital chaplains, and faith leaders. Finally, the diversity of our educators was a major asset, representing all 5 major world religions and the entire spectrum of spirituality, including nonspiritual individuals.

At the same time, there are several areas for future improvement which should be addressed in future investigations. While our course did increase the number of students enrolled from semester 1 to semester 2, we were greatly limited in our analysis by sample size, preventing us from reaching data saturation or performing power calculations. This was primarily due to the elective nature of the course. The course's status as an elective also increases the likelihood of selection bias, with increased spirituality/religiosity among enrolled students causing inflated estimates of course efficacy. To address this concern, we selected a comparison group of medical students from the course waitlist and ensured that both groups were matched with regard to religiosity and spirituality. Nonetheless, full randomization of both groups would allow for more robust conclusions. A future iteration of the course that is integrated as a mandatory part of a medical school curriculum would greatly support this objective.

Another area for future improvement is the course efficacy assessment. While we were able to test levels 1 and 2 of Kirkpatrick's model (reaction and learning), future efforts should aim to assess levels 3 and 4 (behavior and results) to better validate course effectiveness. Such a study would likely need to be performed with higher level medical trainees such as residents since levels 3 and 4 require active utilization of learned skills in actual patient care scenarios, an obstacle for a medical student course. Furthermore, while a major focus of this course was teaching medical students’ communication strategies for effective spiritual care, our aptitude assessment focused primarily on medico-ethical decision-making in the context of a religious/spiritual patient. This was primarily due to the difficulty of creating effective standardized multiple-choice questions for patient care conversations. Proper assessment of such competencies in the future should utilize standardized patients and real-time feedback, something which was not feasible in this study due to the resources required. Finally, our course was taught at a single medical school and thus may not be generalizable to medical students as a whole. Further iterations of the course will be implemented at multiple medical schools, reducing bias. Nonetheless, our study demonstrated strong results given the restrictions imposed by the course format and should serve as proof of the viability of developing an evidence-based spiritual care curriculum.

## Conclusion

Our “Religion and Medicine” course was able to effectively educate students on the principles of effective spiritual care by improving knowledge, attitudes, and confidence regarding S&H fundamentals over the course of the 2023 to 2024 academic year.

## Supplemental Material

sj-docx-1-mde-10.1177_23821205251336846 - Supplemental material for Demonstrating the Viability of Spiritual Care Education: A Pilot Study on Integrating Spirituality and Health into Medical Education Supplemental material, sj-docx-1-mde-10.1177_23821205251336846 for Demonstrating the Viability of Spiritual Care Education: A Pilot Study on Integrating Spirituality and Health into Medical Education by Haafiz Hashim, Zuhair Zaidi, Ahmed Alshaikhsalama, Ammaar Kazi and Zaiba Jetpuri in Journal of Medical Education and Curricular Development

sj-docx-2-mde-10.1177_23821205251336846 - Supplemental material for Demonstrating the Viability of Spiritual Care Education: A Pilot Study on Integrating Spirituality and Health into Medical Education Supplemental material, sj-docx-2-mde-10.1177_23821205251336846 for Demonstrating the Viability of Spiritual Care Education: A Pilot Study on Integrating Spirituality and Health into Medical Education by Haafiz Hashim, Zuhair Zaidi, Ahmed Alshaikhsalama, Ammaar Kazi and Zaiba Jetpuri in Journal of Medical Education and Curricular Development

sj-docx-3-mde-10.1177_23821205251336846 - Supplemental material for Demonstrating the Viability of Spiritual Care Education: A Pilot Study on Integrating Spirituality and Health into Medical Education Supplemental material, sj-docx-3-mde-10.1177_23821205251336846 for Demonstrating the Viability of Spiritual Care Education: A Pilot Study on Integrating Spirituality and Health into Medical Education by Haafiz Hashim, Zuhair Zaidi, Ahmed Alshaikhsalama, Ammaar Kazi and Zaiba Jetpuri in Journal of Medical Education and Curricular Development

## Appendix A

**Course Description: Religion & Medicine ENRH-145**

**Course Directors: Haafiz Hashim and Ahmed Alshaikhsalama, MS2**

**Faculty Sponsor: Dr Zaiba Jetpuri**

**Department: Department of Family Medicine**

**Student Liaison(s): N/A**

**Start Semester/Date: Spring 2023**

### Requirements

Minimum 5 participants for course to be conducted.

Maximum 30 number of students per course (if applicable).

### Rationale

For centuries, various world religions have claimed to offer something of value to the study of health, disease, and healing. From the Judeo-Christian-Islamic tradition to Eastern religions, faith communities have sought to develop rich and meaningful theological beliefs underpinning an understanding of physical well-being and the practice of Medicine. In fact, it could be argued that medicine has its roots in the religiously inclined healers of Ayurveda, traditional Chinese herbal medicine, Qi, and the ancient tribal witch doctors. Furthermore, physicians and patients both approach the healing art of Medicine today having been shaped by a variety of significant worldviews and beliefs. Religious beliefs often play an incredibly formative role in shaping both these worldviews and the expectations surrounding physician–patient encounters within the world of Medicine.

It is therefore of utmost importance that future healthcare practitioners develop an understanding of the wide array of religious beliefs concerning Medicine, not only in order to better understand their patients but also to be able to reflect upon their own role in working to provide healing.

Through dialogue with one another and with religious leaders and physicians from a diverse background of beliefs, we hope to foster a deeper appreciation of the rich and varied history of religious understanding of Medicine and to reflect upon ways that such an appreciation might influence our future practice as culturally competent healthcare professionals.

### Objectives

1. To develop in students an understanding of the religious diversity of their future patients, both in American and global contexts. This is akin to the popular notion of “cultural competency,” but as it concerns religion rather than culture in general.
2. To gain knowledge of the history of the most significant world religions, as well as their core beliefs and worldviews.
3. To gain an understanding of how religious beliefs can and do inform physicians, patients, and their interactions in the healthcare setting.

### Format

- There will be 8 sessions, each 1 to 2 h in length
  - Some sessions will be taught by the course directors
  - Others will be taught by guest speakers from diverse religious backgrounds and the local faith communities
  - Each session will consist of a 40 min to 1.5 h talk on the subject of the week, allowing time for questions, discussion, and orders of business.
- We will provide the opportunity to make up a maximum of 2 classes for each student.
  - One class can be made up by attending a Dallas area or university lecture that in some way deals with the intersection of religion & medicine, and by writing a half-page response to said lecture.
  - One class can be made up by reading an academic article or watching an online video of a lecture or presentation related to religion & medicine, and by writing a half-page response to said media.

### Student Evaluation

Grades will be pass / fail. Attendance is required to receive credit for the course.

### Course Evaluation

Grading will be pass/fail. To receive transcript acknowledgment, students must:

- Receive credit for at least 8 of 9 sessions (OR 7 of 9 sessions and a make-up)
- AND complete the online course evaluation form

### Schedule

Session 01 | Tuesday, January 17th | 6pm

- *Course overview:* Introduction to teaching team. Definitions of spirituality and religion. Introduce whole person care.

Session 02 | Tuesday, January 24th | 6pm

- Overview of religious tenets/history and implications on patient care: Judaism, Christianity, and Islam

Session 03 | Tuesday, January 31st | 6pm

- Overview of religious tenets/history and implications on patient care: Hinduism, Buddhism, and Folk religions

Session 04 | Tuesday, February seventh | 6pm

- Relate experiences with religion and discuss how to interact with those of other faiths

Session 05 | Tuesday, February 14th | 6pm

- *Guest speakers:* Students will have the opportunity to interact with guest speakers that will provide varying perspectives on religion, spirituality, and medicine through the lenses of various faiths

Session 06 | Tuesday, February 21st| 6pm

- Overview of religion and its intersection with medicine in the hospice setting

Session 07 | Tuesday, February 28th | 6pm

- *Case scenarios:* Birth, death, poor prognosis, transfusions, difficult patients, and fasting patients

Session 08 | Tuesday, March 14th | 6pm

- Students will have the opportunity to talk to patients who will share their experiences with medicine and its interaction with their faith

## Appendix B

### Fall 2023 Course Description

Religion & Medicine ENRH-145 is a course that explores the intersection of religion, spirituality, and medicine. Throughout the course, students will engage in discussions with religious leaders, physicians, and patients from various backgrounds to develop cultural competence and gain insights into the historical and contemporary connections between religion and healing.

### Course Directors

Zuhair Zaidi and Ammaar Kazi, MS2

### Faculty Sponsor

Dr Zaiba Jetpuri, Department of Family Medicine

### Start Semester/Date

Fall 2023

### Requirements

- Minimum 5 participants for the course to be conducted
- Maximum 30 students per course (if applicable)

### Rationale

For centuries, world religions have contributed valuable insights to the study of health, disease, and healing. This course aims to provide future healthcare practitioners with an understanding of the religious diversity of their patients and the impact of religious beliefs on healthcare practices. By fostering dialogue and appreciation for different religious perspectives, students will develop cultural competency, which is essential for delivering compassionate and effective healthcare.

### Objectives

1. To develop students’ understanding of the religious diversity of future patients in American and global contexts, focusing on cultural competence related to religion & medicine.
2. To explore the history, core beliefs, and worldviews of the most significant world religions.
3. To understand how religious beliefs inform the perspectives of physicians and patients in the healthcare setting.

### Format

- Eight sessions, each lasting 1 to 2 h.
- Sessions will include presentations by course directors and guest speakers from diverse religious backgrounds and local faith communities.
- Each session will involve a 40-min to 1.5-h talk on the topic of the week, followed by interactive discussions and Q&A.

### Make-Up Opportunities

- Students can make up a maximum of 2 classes:
- One class can be made up by attending a Dallas-area or university lecture related to religion & medicine and writing a half-page response.
- One class can be made up by reading an academic article or watching an online video related to religion & medicine and writing a half-page response.
- *Note:* The ethics bowl is mandatory and cannot be skipped

### Student Evaluation

- Grades will be pass/fail.
- Attendance is required to receive credit for the course.
- All sessions except for the in-person Ethics Bowl will be over zoom!

### Course Evaluation

- Grading will be pass/fail.
- To receive transcript acknowledgment, students must:
- Attend at least 8 sessions (OR 6 sessions and 2 make-up OR 7 Sessions and 1 make-up).
- Complete the online course evaluation form.

### Schedule

Session 01 | Tuesday, August 22nd | 6 pm

- Course Overview, Introduction to teaching team, Definitions of spirituality and religion, Does spirituality/religion affect your health outcomes?

Session 02 | Tuesday, August 29th | 6 pm

- Interview a healthcare Chaplain to speak out his experiences as a Christian in healthcare

Session 03 | Tuesday, September 12th | 6 pm

- Interview a Jewish Person to speak about his experiences in healthcare

Session 04 | Tuesday, September 19th | 6 pm

-Interview a Muslim to speak about his experiences in healthcare

Session 05 | Tuesday, September 26th | 6 pm

- Interview a Hindu priest to speak about his experiences

Session 06 | Tuesday, October third | 6 pm

- Case scenarios: Birth, death, poor prognosis, transfusions, difficult patients, and fasting patients

Session 07 | Tuesday, October 10th | 6 pm

-Ethics Bowl Competition on Religious Scenarios in Medicine, where participants will analyze ethical dilemmas at the intersection of religious beliefs and medical practices, proposing solutions that respect both the patient's religious beliefs and medical ethics principles.

Session 08 | October 18th | 6 pm

- Conclusion and key takeaways and important info for Step 1

### Ethics Bowl

Welcome to the Ethics Bowl Competition on Religious Scenarios in Medicine! In this competition, participants will be presented with various case scenarios that involve ethical dilemmas at the intersection of religious beliefs and medical practices. The participants will be asked to analyze each scenario and propose ethical solutions that respect both the patient's religious beliefs and the principles of medical ethics.

Please note that the scenarios are fictional and designed solely for educational purposes. Let's begin:

Scenario 1: The Blood Transfusion Dilemma

A patient from a devout religious community has been involved in a severe car accident and requires an immediate blood transfusion to save their life. However, the patient's religious beliefs strictly forbid receiving blood transfusions from outside sources. The medical team believes that the blood transfusion is the only way to save the patient's life.

Discuss the ethical considerations involved in this scenario and propose possible solutions that respect the patient's religious beliefs while ensuring their well-being.

Scenario 2: End-of-Life Care and Euthanasia

A terminally ill patient, who is a member of a religious group that firmly opposes euthanasia, is suffering unbearable pain and wishes to end their life through assisted suicide. The medical team acknowledges the patient's pain and the legality of euthanasia in the region.

Explore the ethical challenges faced by the medical team, the patient's family, and society in this situation, and suggest how to navigate these challenges while respecting the patient's religious beliefs.

Scenario 3: In Vitro Fertilization and Religious Beliefs

A couple struggling with infertility opts for in vitro fertilization (IVF) to conceive. However, certain religious beliefs within their community condemn the use of assisted reproductive technologies. The couple firmly believes that IVF is their only chance to have a child.

Examine the ethical implications of this scenario and propose approaches that respect the couple's religious beliefs without compromising the principles of reproductive medicine.

Scenario 4: Adolescent Decision-Making and Religious Autonomy

A 15-year-old patient, who belongs to a religious group with strict dietary restrictions, is diagnosed with a life-threatening condition that requires surgery. The patient's parents, who adhere to the same religious beliefs, refuse the surgery, relying solely on spiritual healing practices.

Analyze the ethical concerns surrounding the autonomy of the adolescent patient, the parental decision-making, and the role of the medical team in such situations. Propose a course of action that respects the patient's religious background while safeguarding their health.

Scenario 5: Cultural and Religious Sensitivity in Patient Care

A Muslim patient is admitted to a hospital and requires medical attention. The patient follows a strict Halal diet, but the hospital's standard meal options do not comply with their dietary requirements. Additionally, the patient requests a private space to pray multiple times a day.

Discuss the ethical considerations of providing culturally and religiously sensitive care to the patient. Offer suggestions on how healthcare providers can respect diverse religious beliefs while maintaining the quality of care.

Remember, in all these scenarios, participants are encouraged to consider the principles of medical ethics, religious freedom, patient autonomy, and cultural sensitivity when proposing solutions.

Best of luck to all participants! Let the Ethics Bowl Competition begin!

Sample Question to Ask Judaism

1. From a religious perspective, how does Judaism view the role of medicine and healthcare in preserving life and promoting well-being?
2. Are there any specific guidelines or teachings within Judaism regarding seeking medical treatment and the use of modern medical advancements?
3. How does the concept of “pikuach nefesh” (saving a life) influence medical decision-making in Judaism?
4. What does Jewish law say about organ transplantation and donation, both in terms of giving and receiving organs?
5. How does Judaism approach the issue of end-of-life care, including topics such as palliative care, hospice, and euthanasia?
6. What advice does Judaism offer to individuals facing ethical dilemmas between adhering to religious beliefs and receiving necessary medical treatments, such as blood transfusions or lifesaving surgeries?
7. In cases where medical procedures conflict with religious observance, how can one strike a balance between respecting religious commitments and ensuring good health?
8. Are there specific dietary guidelines within Judaism that might impact medical treatments, and how can healthcare professionals accommodate these beliefs?
9. How does Judaism view mental health and the use of mental health treatments and therapies?
10. Is there any guidance in Jewish teachings about alternative or complementary medicine practices, and how are these views integrated into the broader healthcare approach?

Remember, the answers to these questions may vary depending on the specific interpretations and practices within different Jewish communities, so it's essential to consider the perspectives of the specific rabbi you are speaking with.
