# Supplementary material for: Demonstrating the Viability of Spiritual Care Education: A Pilot Study on Integrating Spirituality and Health into Medical Education
Source: J Med Educ Curric Dev. 2025 May 15;12:23821205251336846. doi: 10.1177/23821205251336846 (PMC12081970; doi:10.1177/23821205251336846)
Supplement: sj-docx-1-mde-10.1177_23821205251336846 - Supplemental material for Demonstrating the Viability of Spiritual Care Education: A Pilot Study on Integrating Spirituality and Health into Medical Education [file sj-docx-1-mde-10.1177_23821205251336846.docx]

**Course Description: Religion & Medicine ENRH-145**

**Course Directors: Haafiz Hashim and Ahmed Alshaikhsalama, MS2**

**Faculty Sponsor: Dr. Zaiba Jetpuri**

**Department: Department of Family Medicine**

**Student Liaison(s): N/A**

**Start Semester/Date: Spring 2023**

**Requirements:**

5 Minimum participants for course to be conducted

30 Maximum number of students per course (if applicable)

**Rationale:**

For centuries, various world religions have claimed to offer something of value to the study of health, disease, and healing. From the Judeo-Christian-Islamic tradition to Eastern religions, faith communities have sought to develop rich and meaningful theological beliefs underpinning an understanding of physical well-being and the practice of Medicine. In fact, it could be argued that medicine has its roots in the religiously inclined healers of Ayurveda, traditional Chinese herbal medicine, Qi, and the ancient tribal witch doctors. Furthermore, physicians and patients today both approach the healing art of Medicine having been shaped by a variety of significant worldviews and beliefs. Religious beliefs often play an incredibly formative role in shaping both these worldviews and the expectations surrounding physician-patient encounters within the world of Medicine.

It is therefore of utmost importance that future healthcare practitioners develop an understanding of the wide array of religious beliefs concerning Medicine, not only in order to better understand their patients, but also to be able to reflect upon their own role in working to provide healing.

Through dialogue with one another and with religious leaders and physicians from a diverse background of beliefs, we hope to foster a deeper appreciation of the rich and varied history of religious understanding of Medicine and to reflect upon ways that such an appreciation might influence our future practice as culturally competent healthcare professionals.

**Objectives:**

1. To develop in students an understanding of the religious diversity of their future patients, both in American and global contexts. This is akin to the popular notion of “cultural competency,” but as it concerns religion rather than culture in general.

2. To gain knowledge of the history of the most significant world religions, as well as their core beliefs and worldviews.

3. To gain an understanding of how religious beliefs can and do inform physicians, patients, and their interactions in the healthcare setting.

**Format**

• There will be 8 sessions, each 1-2 hours in length

o Some sessions will be taught by the course directors

o Others will be taught by guest speakers from diverse religious backgrounds and the local faith communities

o Each session will consist of a 40 min – 1.5 hour talk on the subject of the week, allowing time for questions, discussion, and orders of business.

• We will provide the opportunity to make up a maximum of two classes for each student. o One class can be made up by attending a Dallas-area or university lecture that in some way deals with the intersection of religion & medicine and by writing a half-page response to said lecture.

o One class can be made up by reading an academic article or watching an online video of a lecture or presentation related to religion & medicine, and by writing a half-page response to said media.

**Student Evaluation**

Grades will be pass / fail. Attendance is required to receive credit for the course. **Course Evaluation**

Grading will be pass/fail. To receive transcript acknowledgment, students must:

• Receive credit for at least 8 of 9 sessions (OR 7 of 9 sessions and a make-up) • AND complete the online course evaluation form

**Schedule**

Session 01 | Tuesday, January 17th | 6pm

• Course Overview. Introduction to teaching team. Definitions of spirituality and religion. Introduce whole person care.

Session 02 | Tuesday, January 24th | 6pm

• Overview of religious tenets/history and implications on patient care: Judaism, Christianity, Islam

Session 03 | Tuesday, January 31st | 6pm

• Overview of religious tenets/history and implications on patient care: Hinduism, Buddhism, Folk religions

Session 04 | Tuesday, February 7th | 6pm

• Relate experiences with religion and discuss how to interact with those of other faiths Session 05 | Tuesday, February 14th | 6pm

• Guest speakers: students will have the opportunity to interact with guest speakers that will provide varying perspectives on religion, spirituality, and medicine through the lenses of various faiths

Session 06 | Tuesday, February 21st| 6pm

• Overview of religion and its intersection with medicine in the hospice setting Session 07 | Tuesday, February 28th | 6pm

• Case scenarios: birth, death, poor prognosis, transfusions, difficult patients, and fasting patients Session 08 | Tuesday, March 14th | 6pm

• Students will have the opportunity to talk to patients who will share their experiences with medicine and its interaction with their faith
