# Supplementary material for: Demonstrating the Viability of Spiritual Care Education: A Pilot Study on Integrating Spirituality and Health into Medical Education
Source: J Med Educ Curric Dev. 2025 May 15;12:23821205251336846. doi: 10.1177/23821205251336846 (PMC12081970; doi:10.1177/23821205251336846)
Supplement: sj-docx-2-mde-10.1177_23821205251336846 - Supplemental material for Demonstrating the Viability of Spiritual Care Education: A Pilot Study on Integrating Spirituality and Health into Medical Education [file sj-docx-2-mde-10.1177_23821205251336846.docx]

**Fall 2023 Course Description:**

Religion & Medicine ENRH-145 is a course that explores the intersection of religion, spirituality, and medicine. Throughout the course, students will engage in discussions with religious leaders, physicians, and patients from various backgrounds to develop cultural competence and gain insights into the historical and contemporary connections between religion and healing.

**Course Directors:**

Zuhair Zaidi and Ammaar Kazi, MS2

**Faculty Sponsor:**

Dr. Zaiba Jetpuri, Department of Family Medicine

**Start Semester/Date:**

Fall 2023

**Requirements:**

- Minimum 5 participants for the course to be conducted
- Maximum 30 students per course (if applicable)

**Rationale:**

For centuries, world religions have contributed valuable insights to the study of health, disease,

and healing. This course aims to provide future healthcare practitioners with an understanding of the religious diversity of their patients and the impact of religious beliefs on healthcare practices. By fostering dialogue and appreciation for different religious perspectives, students will develop cultural competency, which is essential for delivering compassionate and effective healthcare.

**Objectives:**

1. To develop students' understanding of the religious diversity of future patients in American and global contexts, focusing on cultural competence related to religion and medicine.

2. To explore the history, core beliefs, and worldviews of the most significant world religions.

3. To understand how religious beliefs inform the perspectives of physicians and patients in the healthcare setting.

**Format:**

- Eight sessions, each lasting 1-2 hours.
- Sessions will include presentations by course directors and guest speakers from diverse religious backgrounds and local faith communities.
- Each session will involve a 40-minute to 1.5-hour talk on the topic of the week, followed by interactive discussions and Q&A.

**Make-Up Opportunities:**

- Students can make up a maximum of two classes:
- One class can be made up by attending a Dallas-area or university lecture related to religion & medicine and writing a half-page response.
- One class can be made up by reading an academic article or watching an online video related to religion & medicine and writing a half-page response.
- Note: The ethics bowl is mandatory and cannot be skipped

**Student Evaluation:**

- Grades will be pass/fail.
- Attendance is required to receive credit for the course.
- All sessions except for the in-person Ethics Bowl will be over zoom!

**Course Evaluation:**

- Grading will be pass/fail.
- To receive transcript acknowledgment, students must:
- Attend at least 8 sessions (OR 6 sessions and 2 make-up OR 7 Sessions and 1 make-up).
- Complete the online course evaluation form.

**Schedule:**

Session 01 | Tuesday, August 22nd | 6 pm

- Course Overview, Introduction to teaching team, Definitions of spirituality and religion, Does spirituality/religion affect your health outcomes?

Session 02 | Tuesday, August 29th | 6 pm

- Interview a healthcare Chaplain to speak out his experiences as a Christian in healthcare

Session 03 | Tuesday, September 12th | 6 pm

- Interview a Jewish Person to speak about his experiences in healthcare

Session 04 | Tuesday, September 19th | 6 pm

-Interview a Muslim to speak about his experiences in healthcare

Session 05 | Tuesday, September 26th | 6 pm

- Interview a Hindu priest to speak about his experiences

Session 06 | Tuesday, October 3rd | 6 pm

- Case scenarios: Birth, death, poor prognosis, transfusions, difficult patients, and fasting patients

Session 07 | Tuesday, October 10th | 6 pm

-Ethics Bowl Competition on Religious Scenarios in Medicine, where participants will analyze ethical dilemmas at the intersection of religious beliefs and medical practices, proposing solutions that respect both the patient's religious beliefs and medical ethics principles.

Session 08 | October 18th | 6 pm

- Conclusion and key takeaways and important info for Step 1

**Ethics Bowl**

Welcome to the Ethics Bowl Competition on Religious Scenarios in Medicine! In this competition, participants will be presented with various case scenarios that involve ethical dilemmas at the intersection of religious beliefs and medical practices. The participants will be asked to analyze each scenario and propose ethical solutions that respect both the patient's religious beliefs and the principles of medical ethics.

Please note that the scenarios are fictional and designed solely for educational purposes. Let's begin:

Scenario 1: The Blood Transfusion Dilemma

A patient from a devout religious community has been involved in a severe car accident and requires an immediate blood transfusion to save their life. However, the patient's religious beliefs strictly forbid receiving blood transfusions from outside sources. The medical team believes that the blood transfusion is the only way to save the patient's life.

Discuss the ethical considerations involved in this scenario and propose possible solutions that respect the patient's religious beliefs while ensuring their well-being.

Scenario 2: End-of-Life Care and Euthanasia

A terminally ill patient, who is a member of a religious group that firmly opposes euthanasia, is suffering unbearable pain and wishes to end their life through assisted suicide. The medical team acknowledges the patient's pain and the legality of euthanasia in the region.

Explore the ethical challenges faced by the medical team, the patient's family, and society in this situation, and suggest how to navigate these challenges while respecting the patient's religious beliefs.

Scenario 3: In Vitro Fertilization and Religious Beliefs

A couple struggling with infertility opts for in vitro fertilization (IVF) to conceive. However, certain religious beliefs within their community condemn the use of assisted reproductive technologies. The couple firmly believes that IVF is their only chance to have a child.

Examine the ethical implications of this scenario and propose approaches that respect the couple's religious beliefs without compromising the principles of reproductive medicine.

Scenario 4: Adolescent Decision-Making and Religious Autonomy

A 15-year-old patient, who belongs to a religious group with strict dietary restrictions, is diagnosed with a life-threatening condition that requires a surgery. The patient's parents, who adhere to the same religious beliefs, refuse the surgery, relying solely on spiritual healing practices.

Analyze the ethical concerns surrounding the autonomy of the adolescent patient, the parental decision-making, and the role of the medical team in such situations. Propose a course of action that respects the patient's religious background while safeguarding their health.

Scenario 5: Cultural and Religious Sensitivity in Patient Care

A Muslim patient is admitted to a hospital and requires medical attention. The patient follows a strict Halal diet, but the hospital's standard meal options do not comply with their dietary requirements. Additionally, the patient requests a private space to pray multiple times a day.

Discuss the ethical considerations of providing culturally and religiously sensitive care to the patient. Offer suggestions on how healthcare providers can respect diverse religious beliefs while maintaining the quality of care.

Remember, in all these scenarios, participants are encouraged to consider the principles of medical ethics, religious freedom, patient autonomy, and cultural sensitivity when proposing solutions.

Best of luck to all participants! Let the Ethics Bowl Competition begin!

Sample Question to Ask Judaism

1. From a religious perspective, how does Judaism view the role of medicine and healthcare in preserving life and promoting well-being?

2. Are there any specific guidelines or teachings within Judaism regarding seeking medical treatment and the use of modern medical advancements?

3. How does the concept of "pikuach nefesh" (saving a life) influence medical decision-making in Judaism?

4. What does Jewish law say about organ transplantation and donation, both in terms of giving and receiving organs?

5. How does Judaism approach the issue of end-of-life care, including topics such as palliative care, hospice, and euthanasia?

6. What advice does Judaism offer to individuals facing ethical dilemmas between adhering to religious beliefs and receiving necessary medical treatments, such as blood transfusions or life-saving surgeries?

7. In cases where medical procedures conflict with religious observance, how can one strike a balance between respecting religious commitments and ensuring good health?

8. Are there specific dietary guidelines within Judaism that might impact medical treatments, and how can healthcare professionals accommodate these beliefs?

9. How does Judaism view mental health and the use of mental health treatments and therapies?

10. Is there any guidance in Jewish teachings about alternative or complementary medicine practices, and how are these views integrated into the broader healthcare approach?

Remember, the answers to these questions may vary depending on the specific interpretations and practices within different Jewish communities, so it's essential to consider the perspectives of the specific rabbi you are speaking with.
